# Antimalarial drugs: unexpected evolutionary consequences

**DOI:** 10.1186/1475-2875-9-S2-P45

**Published:** 2010-10-20

**Authors:** Petra Schneider, Andy S Bell, Andrew F Read, Sarah E Reece

**Affiliations:** 1 Centre for Immunity, Infection and Evolution, School of Biological Sciences, University of Edinburgh, Edinburgh, EH9 3JT, UK; 2 Centre for Infectious Disease Dynamics, Departments of Biology and Entomology, Pennsylvania State University, University Park, Pennsylvania, USA; 3 Institutes of Evolution, Immunology and Infection Research, School of Biological Sciences, University of Edinburgh, Edinburgh, EH9 3JT, UK

## Background

For malaria parasites, exposure to drugs is now an integral, human-induced, part of their ecology that exerts substantial selective effects on their traits. Currently used drugs often do not eliminate transmission, which enables evolution of traits that are beneficial for parasites. The appearance and spread of resistance mutations that protect parasites through direct interactions with drug molecules are well documented and often assumed to underlie treatment failures. However, this is just one example of how parasites can minimise their vulnerability to drugs. Other parasite traits (e.g. growth rate, conversion rate, red blood cell preference) that parasites have evolved independently of drugs can also provide indirect protection from drugs. Both lab and field data suggest that such traits can influence parasite survival and transmission in drug treated malaria infections.

## Methods

We used a rodent malaria model *(Plasmodium chabaudi)* to test if virulence can confer protection to parasites in drug-treated infections. Mice were infected with genetically similar virulent or avirulent genotypes (CWvir orCWavir), with or without an additional competing genotype (DK). Parasites were then exposed to drugs (control, pyrimethamine or artemisinin, across several doses and treatment regimes) and we compared parasite survival, production of gametocytes and, in a subset of infections, transmission to mosquitoes.

## Results

We show virulent parasites better survived treatment with pyrimethamine^1^ or artemisinin, and produce more gametocytes than genetically related avirulent parasites. The survival and gametocyte production benefits for the virulent parasites held fora range of drug doses, duration of treatment and for drugs with different modes of action.

## Conclusions

Drug sensitivity can be virulence dependent, leading to a survival and transmission advantage for virulent parasites. If these results are general across parasite genotypes and species, this has consequences for the evolutionary trajectories of parasites resulting from continued population-wide exposure to drugs^2^. I will discuss how to overcome the challenge of translating our laboratory findings to natural infections in humans and how to integrate our epidemiological and evolutionary findings into a framework for malaria control.

## References

[B1] Schneider P Chan BH Reece SE Read AF Does the drug sensitivity of malaria parasites depend on their virulence? Malaria Journal 2008 7 257 10.1186/1475-2875-7-257 19087299 PMC2636820

[B2] Stein WD Sanchez CP Lanzer M Virulence and drug resistance in malaria parasites Trends in Parasitology 2009 25 441 443 10.1016/j.pt.2009.07.003 19734096

